# Multimodal imaging and clinical evolution of an optic nerve head granuloma with prominent overlying vessels in ocular sarcoidosis: a case report and literature review

**DOI:** 10.3389/fneur.2026.1978059

**Published:** 2026-10-15

**Authors:** Jiahao Xiong, Jiyue Tan, Jun Xiao

**Affiliations:** Department of Ophthalmology, The Second Hospital of Jilin University, Changchun, China

**Keywords:** case report, multimodal imaging, ocular sarcoidosis, optic nerve head granuloma, optical coherence tomography angiography

## Abstract

**Background:**

Sarcoidosis is a multisystem granulomatous disorder of unclear etiology, histopathologically characterized by noncaseating granulomas. Ocular involvement is among the most common extrapulmonary manifestations, whereas optic nerve involvement occurs in fewer than 5% of cases. Direct granulomatous infiltration of the optic nerve represents the rarest form of optic nerve involvement. We report a rare case of a sarcoidosis-associated optic nerve head granuloma with prominent overlying vessels and describe its multimodal imaging features, clinical course, and treatment response, with a review of the clinical spectrum of sarcoid optic nerve head granulomas reported in the English-language literature.

**Case presentation:**

A 31-year-old woman presented with floaters in the left eye. Best-corrected visual acuity was 20/20 in both eyes. Fundus examination of the left eye revealed a white elevated optic nerve head lesion with fine tortuous surface vessels, accompanied by vitreous inflammatory haze, retinal periphlebitis, and candle-wax drippings. Fundus fluorescein angiography demonstrated early filling of lesion-associated vessels with marked late leakage, and optical coherence tomography angiography detected abnormal flow signals corresponding to the lesion. Additionally, chest computed tomography revealed bilateral hilar lymphadenopathy, and transbronchial lung biopsy demonstrated chronic granulomatous inflammation with multinucleated giant cells and no definite necrosis. The overall findings supported the diagnosis of sarcoidosis. The lesion initially progressed after the patient declined corticosteroid therapy, but gradually regressed following systemic corticosteroid treatment, with parallel disappearance of the lesion-associated abnormal flow signals. At the final follow-up, the lesion had completely regressed, leaving only a thin gliotic membrane, while visual acuity remained 20/20 throughout the entire follow-up.

**Conclusion:**

Although rare, sarcoid optic nerve head granuloma is a highly suggestive ocular manifestation that may be the presenting sign of systemic sarcoidosis and can mimic infectious or neoplastic optic disc mass lesions. Multimodal imaging is valuable for early recognition, assessment of treatment response, and longitudinal monitoring. Once the diagnosis is established, corticosteroid-based systemic therapy may be considered. In this case, systemic corticosteroid therapy was associated with anatomic regression of the optic nerve head granuloma, while visual acuity remained stable throughout follow-up.

## Background

Sarcoidosis is a multisystem inflammatory disorder of unknown etiology, histopathologically characterized by non-necrotizing granuloma formation, with the lungs being the most commonly affected organ (1). Ocular involvement is among the most common extrathoracic manifestations of sarcoidosis and has been reported in 10–55% of patients (2). Neurosarcoidosis occurs in approximately 5–15% of individuals with the disease; however, involvement of the anterior visual pathway—including the optic nerve, optic chiasm, and optic tract—is uncommon, occurring in only 1–5% of patients with sarcoidosis (3).

Herein, we report a rare case of sarcoidosis in which an optic nerve head granuloma was the presenting sign. We highlight its multimodal imaging features, progression in the absence of treatment, and serial evolution after systemic corticosteroid therapy. We also review the English-language literature and summarize 48 previously reported cases of sarcoid optic nerve head granuloma. To the best of our knowledge, this represents the largest English-language summary to date of cases presenting with this distinctive fundus finding.

## Case presentation

In February 2024, a 31-year-old woman presented with a 2-day history of floaters in the left eye. She reported no known systemic disease and no family history of inherited disorders. Best-corrected visual acuity (BCVA) was 20/20 in both eyes. No clinically significant abnormalities were noted in the right eye. Slit-lamp examination of the left eye showed a quiet anterior segment with mild vitreous haze. Fundus examination of the left eye revealed a white mass at the inferior aspect of the optic disc, partially obscuring the disc. The lesion, measuring approximately 1 disc diameter, had fine tortuous surface vessels. Additionally, retinal periphlebitis with candle-wax drippings was noted inferior to the disc, together with vascular sheathing in the inferotemporal peripheral retina (Figure 1A). Optical coherence tomography (OCT) through the lesion demonstrated an elevated mass protruding into the vitreous cavity with posterior shadowing (Figure 1B). Fundus fluorescein angiography (FFA) showed early filling of the tortuous surface vessels overlying the lesion with mild leakage in the early phase, progressing to marked leakage, accompanied by staining of both the lesion and the optic disc in the late phase; perivascular leakage along the inferotemporal branches was also noted (Figures 1C–E). Optical coherence tomography angiography (OCTA) showed lesion-associated abnormal flow signals within the vitreous slab (Figure 1F).

**Figure 1:**
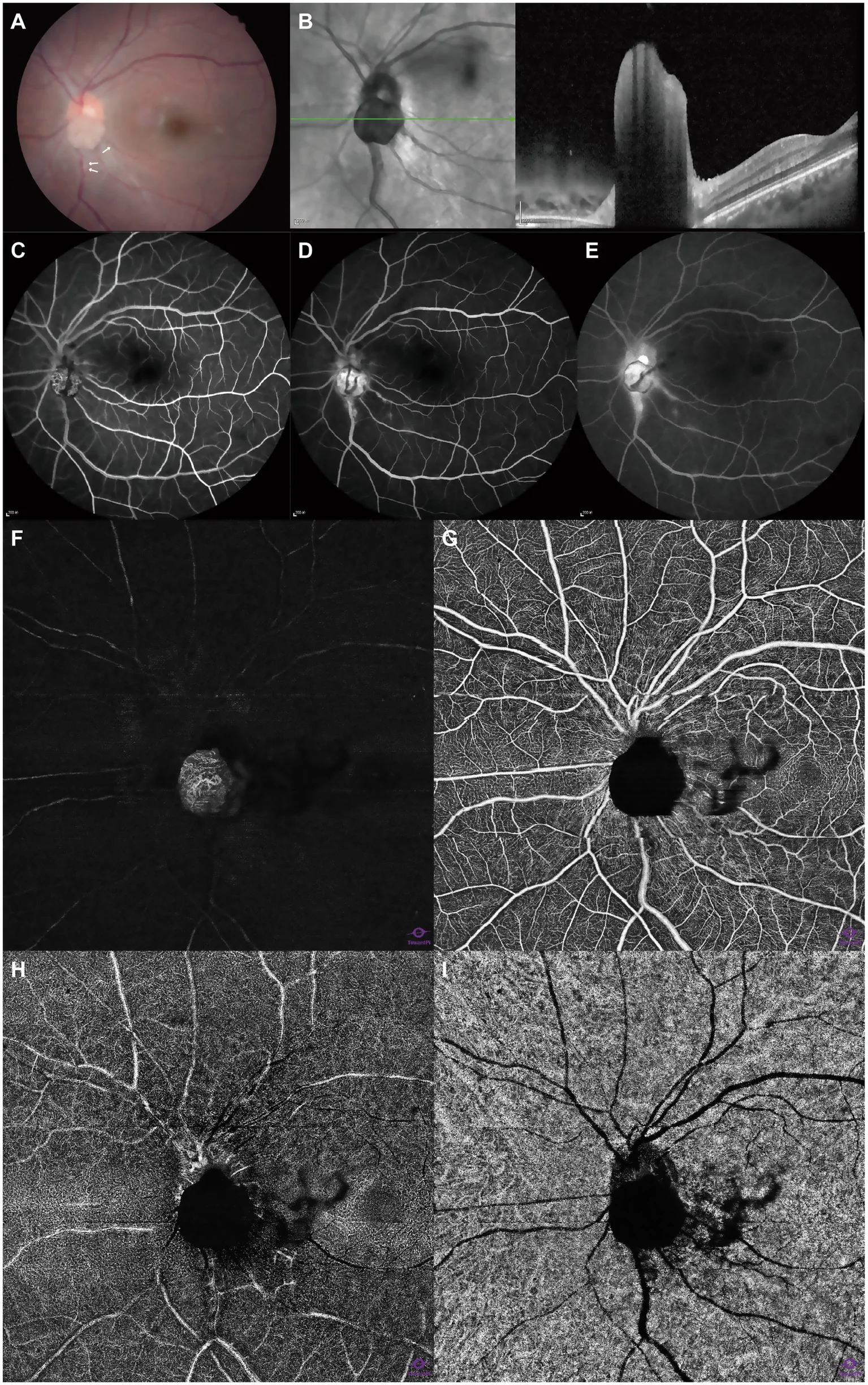
Baseline multimodal imaging findings. **(A)** Color fundus photograph of the left eye at presentation, showing a white mass approximately 1 disc diameter in size overlying the optic disc, with tortuous small vessels on its surface. Perivascular candle-wax drippings are visible inferior to the disc (arrows). **(B)** Spectral-domain optical coherence tomography (OCT) image showing a hyperreflective elevated lesion with posterior shadowing. **(C, D)** Early-phase fundus fluorescein angiography (FFA) of the left eye showing early filling of tortuous vessels overlying the lesion, with mild leakage.**(E)** Late-phase FFA demonstrating marked leakage from the lesion, staining of the lesion and optic disc, and vessel wall leakage in the inferotemporal branch retinal vessels. **(F–I)** OCTA images of the same lesion at different segmentation levels. F shows the vitreous slab, G the superficial capillary plexus (SCP), H the deep capillary plexus (DCP), and I the choriocapillaris (CC). Lesion-associated abnormal flow signals are detected in the vitreous slab.

Systemic evaluation was subsequently undertaken. Review of the complete non-contrast chest computed tomography (CT) series (1-mm slice thickness) demonstrated multiple bilateral pulmonary nodules, mediastinal lymphadenopathy, and bilateral hilar lymphadenopathy. Representative CT images and the corresponding chest CT report are provided in Supplementary Figure 1. Histopathologic examination of a transbronchial lung biopsy (TBLB) specimen from the right middle lobe demonstrated chronic granulomatous inflammation with multinucleated giant cells and no definite necrosis (Supplementary Figure 2). The findings raised the possibility of sarcoidosis or another granulomatous disorder in the appropriate clinical context. In addition, examination of bronchoalveolar lavage fluid (BALF) obtained from the right middle lobe revealed no acid-fast bacilli or malignant cells. Given the left optic nerve head infiltration, magnetic resonance imaging (MRI) of the optic nerves and brain was performed to exclude central nervous system (CNS) or intraorbital optic nerve involvement; no definite abnormality was detected. Systemic corticosteroid therapy was recommended, but the patient initially declined treatment.

At follow-up in May 2024, the left eye showed vitreous snowballs, enlargement of the optic nerve head granuloma, and increased candle-wax drippings (Figure 2A). FFA demonstrated diffuse leakage from the peripheral retinal vessels, consistent with active retinal vasculitis (Figure 2B). In June 2024, the patient was admitted to the Department of Respiratory Medicine for further systemic evaluation and treatment. Serologic testing for hepatitis B, hepatitis C, syphilis, and HIV was negative. As part of the evaluation for infectious causes of granulomatous disease, sputum cultures yielded no pathogenic bacteria or fungi, and no acid-fast bacilli were detected on concentrated sputum examination. The patient received intravenous methylprednisolone at 40 mg/day from June 6 to June 9, followed by 20 mg/day from June 10 to June 12, for a total of 7 days of intravenous corticosteroid therapy. After discharge, treatment was transitioned to oral prednisone at 35 mg/day for the first 2 weeks, followed by 30 mg/day for 1 month. The dose was subsequently reduced to 25 mg/day and thereafter tapered by 5 mg every 2 weeks according to the clinical response. Low-dose prednisone at 5 mg/day was maintained during the later phase of treatment before eventual discontinuation. The total duration of systemic corticosteroid therapy was approximately 16 months. No clinical relapse occurred during corticosteroid tapering or throughout the treatment period.

**Figure 2:**
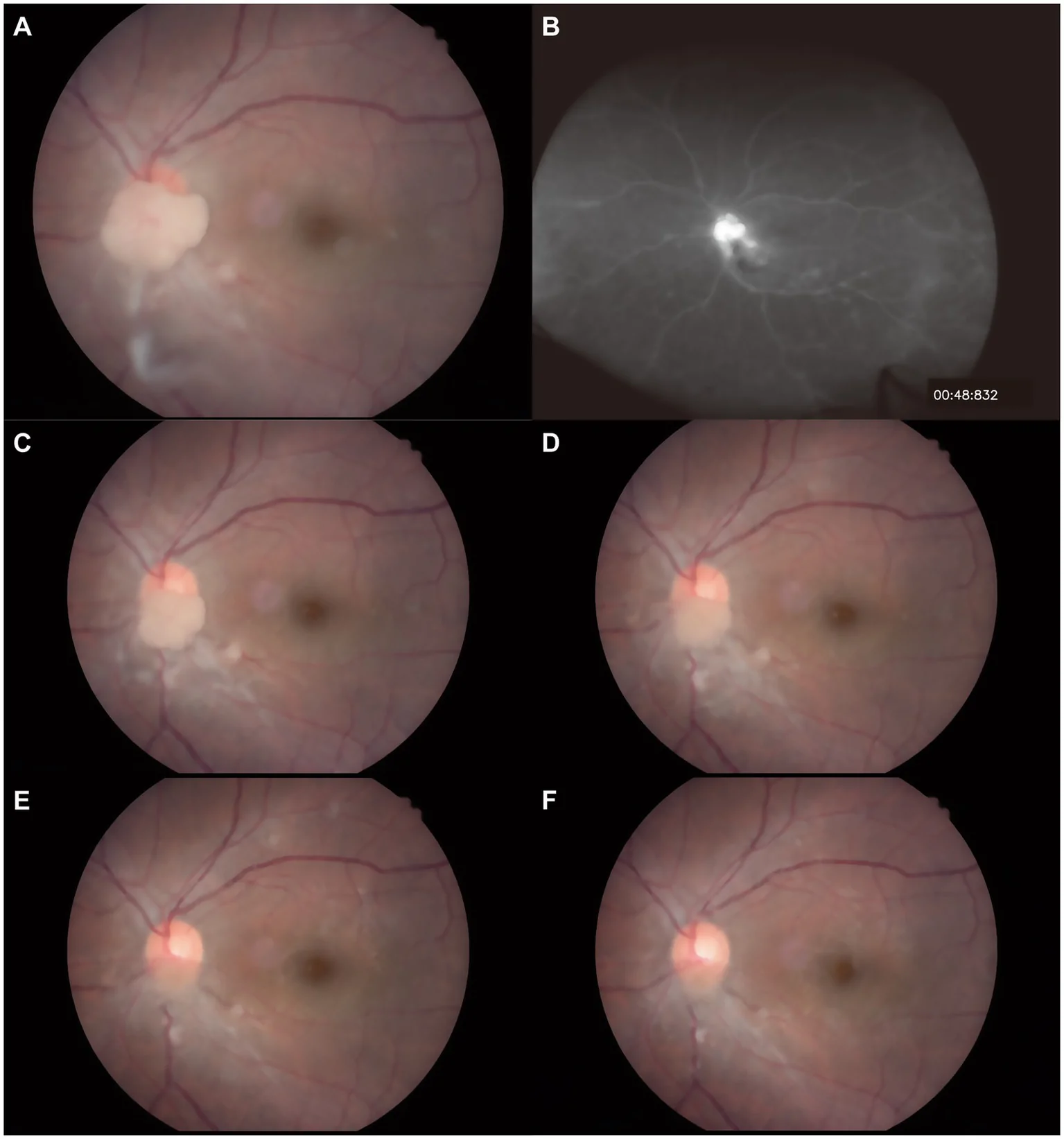
Longitudinal multimodal imaging evolution before and after treatment. Follow-up imaging in May 2024. **(A)** Color fundus photograph of the left eye showing enlargement of the optic disc lesion with increased perivascular candle-wax drippings. **(B)** FFA demonstrating extensive leakage from peripheral retinal vessels. Color fundus photographs of the left eye obtained at 1 month **(C)**, 3 months **(D)**, 6 months **(E)** and 12 months **(F)** after treatment initiation, showing progressive regression of the optic nerve head granuloma with gradual disappearance of the associated abnormal vessels.

Over the course of follow-up, the left optic nerve head granuloma and its associated abnormal vessels progressively regressed (Figures 2C–F). Repeat OCTA in February 2025 demonstrated complete disappearance of the abnormal flow signals corresponding to the lesion (Figures 3A–D). At the final follow-up in October 2025, the lesion had completely regressed, leaving only a thin gliotic membrane (Figure 3E). Throughout the entire course, BCVA remained 20/20 in both eyes. The clinical timeline for the patient was summarized in Table 1.

**Figure 3:**
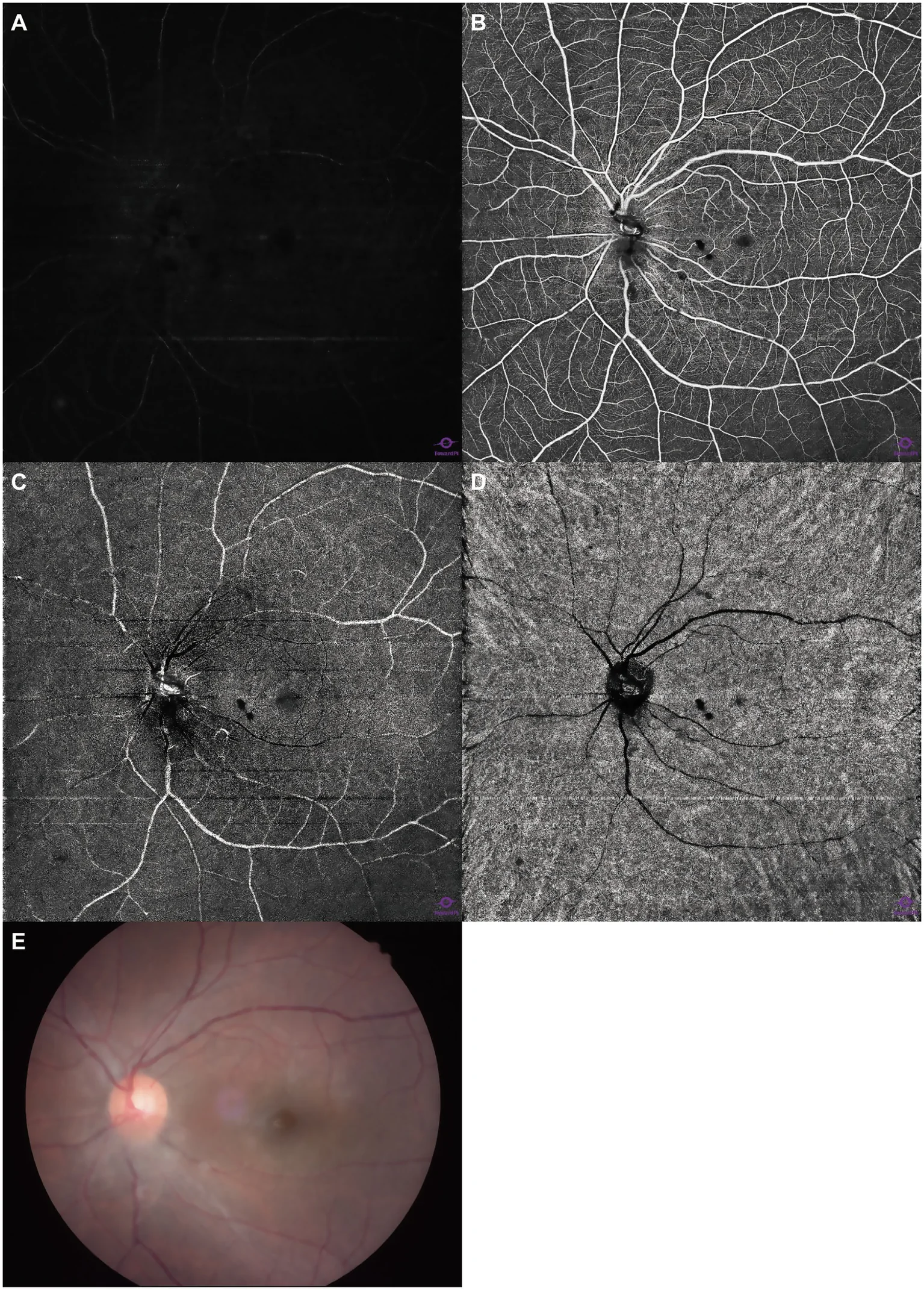
Multimodal imaging findings at the last follow-up. Follow-up OCTA of the left eye obtained in February 2025. **(A–D)** OCTA images segmented at the vitreous, superficial capillary plexus (SCP), deep capillary plexus (DCP), and choriocapillaris (CC) levels, respectively, showing disappearance of the abnormal flow signals corresponding to the lesion. **(E)** Color fundus photograph of the left eye at the last follow-up in October 2025, showing complete regression of the lesion, with only a thin residual gliotic membrane remaining.

**Table 1:** Timeline of the clinical course.

Time point	Key clinical events and findings	Interventions and management
Initial presentation (February 2024)	BCVA was 20/20 in both eyes. Fundus examination showed an optic nerve head granuloma with prominent vessels overlying its surface, accompanied by mild vitreous haze, retinal periphlebitis, and candle-wax drippings. FFA demonstrated early filling of lesion-associated vessels with marked late leakage, and OCTA detected lesion-associated abnormal flow signals.	Systemic evaluation was initiated. No treatment was started at the initial visit.
Initial systemic work-up	Chest CT revealed multiple bilateral pulmonary nodules, mediastinal lymphadenopathy, and bilateral hilar lymphadenopathy. TBLB showed chronic granulomatous inflammation with multinucleated giant cells and no definite necrosis. Examination of bronchoalveolar lavage fluid (BALF) obtained from the right middle lobe revealed no acid-fast bacilli or malignant cells.	Systemic corticosteroid therapy was recommended but initially declined by the patient.
May 2024	Follow-up examination showed enlargement of the optic nerve head granuloma, vitreous snowballs, and increased candle-wax drippings. FFA demonstrated diffuse peripheral retinal vascular leakage, consistent with active retinal vasculitis.	Disease progression before treatment was documented.
June 2024	Serologic testing for hepatitis B, hepatitis C, syphilis, and HIV was negative. As part of the evaluation for infectious causes of granulomatous disease, sputum cultures yielded no pathogenic bacteria or fungi, and no acid-fast bacilli were detected on concentrated sputum examination.	Intravenous methylprednisolone was administered, followed by oral corticosteroids with gradual tapering.
1–12 months after treatment initiation	Serial fundus photographs showed progressive regression of the optic nerve head granuloma and gradual disappearance of the associated abnormal vessels.	Corticosteroid therapy was continued and tapered according to clinical response.
February 2025	Follow-up OCTA showed complete disappearance of the abnormal flow signals corresponding to the previous lesion.	Continued observation was performed to monitor lesion activity.
Final follow-up (October 2025)	The lesion had completely regressed, leaving only a thin residual gliotic membrane. BCVA remained 20/20 throughout follow-up.	No additional ocular intervention was required.

BALF, bronchoalveolar lavage fluid; BCVA, best-corrected visual acuity; CT, computed tomography; FFA, fundus fluorescein angiography; OCTA, optical coherence tomography angiography; TBLB, transbronchial lung biopsy.

### Narrative review of the literature

Previously reported cases of sarcoid optic nerve head granuloma in the English-language literature are summarized in Supplementary Table 1 (4–39). To inform this narrative review, a targeted literature search was performed in PubMed from database inception to May 1, 2026, using the terms (“optic disc granuloma” OR “optic nerve head granuloma” OR “peripapillary granuloma”) AND (“sarcoidosis” OR “sarcoid”). Google Scholar was used as a supplementary source to identify potentially relevant reports not retrieved through PubMed. Reference lists of relevant publications were also examined for additional reports.

The review focused on English-language case reports and case series containing patient-level clinical information. Correspondence, letters, and response articles were also considered when they contained original patient-level data relevant to sarcoid optic nerve head granuloma. These publication types were retained because rare or unusual ophthalmic presentations may be reported in abbreviated formats rather than as full-length case reports. Correspondence or response articles containing only commentary, without original clinical information, were not used as independent case sources.

To minimize duplicate counting, all potentially relevant publications were cross-checked at the patient level. Reports were compared using authorship, institution or geographic location, publication chronology, patient age and sex, laterality, clinical presentation, associated ocular or systemic findings, treatment regimen, follow-up information, and citations to earlier reports when available. When two or more publications were judged to describe the same patient, they were counted as a single case, represented by the report providing the most complete clinical description.

After patient-level cross-checking, 48 distinct previously reported patients were identified, with reports dating back to 1944. Together with the present case, these patients are summarized descriptively in Supplementary Table 1, which includes 49 patients and 57 affected eyes.

Among the 49 patients summarized in Supplementary Table 1, including the present case, ages ranged from 16 to 76 years, with a nearly equal sex distribution (24 male and 25 female patients). Race was inconsistently reported; among the 21 patients for whom this information was available, 11 were Black, 6 were White, and 4 were Asian. Unilateral optic nerve head granuloma was observed in 41 patients, whereas bilateral involvement was present in 8 patients. Notably, 39 of the 49 patients had no previous diagnosis of sarcoidosis, with optic nerve head granuloma serving as the presenting sign of the disease.

Treatment information was available for 44 patients. Corticosteroids were the cornerstone of therapy and were used in all 40 treated patients. Four early cases received no treatment. Corticosteroid monotherapy was used in 25 patients, including systemic, periocular, and topical formulations. Combination therapy with corticosteroids and additional immunosuppressive agents was reported in 12 patients. Methotrexate was the most commonly used steroid-sparing agent, administered in 10 cases; other agents included mycophenolate mofetil and azathioprine. Four patients received biologic therapy, including infliximab, adalimumab, and rituximab, reflecting contemporary treatment approaches for complex or refractory cases. Additionally, two patients underwent intravitreal dexamethasone implantation as part of their overall management.

The clinical course and outcomes of optic nerve head granulomas were documented in 30 patients. Most demonstrated either complete or partial regression following treatment, whereas a smaller number remained stable or showed progression. Complete regression was described in 13 patients and partial regression in 11, while 3 patients remained stable and 3 demonstrated progression. Final visual acuity was reported for 40 affected eyes and varied considerably: 13 eyes achieved 20/20 or better, whereas 12 eyes had final visual acuity of 20/200 or worse. Poor visual outcomes were often reported in eyes with substantial concomitant ocular disease, including severe uveitis, retinal detachment, central retinal vein occlusion, macular edema, and secondary glaucoma. Given the case-based nature of the available literature, however, the relative contribution of the optic nerve head granuloma itself versus these accompanying complications to final visual outcome cannot be determined.

Because the available literature consists predominantly of individual case reports and small case series, including some reports published in correspondence formats, the completeness and level of clinical detail varied substantially among publications. These observations should therefore be interpreted as a descriptive summary of published experience rather than estimates of disease frequency, treatment effectiveness, or prognosis in the broader population. The literature is also subject to incomplete reporting and potential publication and selection bias.

## Discussion

Ocular sarcoidosis can affect virtually any ocular or adnexal structure, giving rise to a broad spectrum of clinical manifestations. Uveitis is the most common ocular manifestation, although the conjunctiva, sclera, cornea, lacrimal gland, orbit, and optic nerve may also be involved (40). Optic nerve involvement, however, is relatively uncommon, with various forms of optic neuropathy reported in approximately 1–5% of patients with sarcoidosis (3). Ingestad and Stigmar classified optic nerve involvement in sarcoidosis into five categories: optic disc edema secondary to posterior uveitis; optic disc edema secondary to increased intracranial pressure; retrobulbar optic neuritis; optic atrophy secondary to compression or infiltration by primary CNS lesions; and primary optic nerve granuloma (7).

Optic nerve head granuloma lies at the interface between ocular sarcoidosis and neurosarcoidosis. Although rare, it is a highly suggestive and clinically important finding. In the present study, we focus on optic nerve head granuloma secondary to sarcoidosis. Because the differential diagnosis of mass-like lesions involving the optic nerve head and peripapillary region is broad, other infiltrative optic neuropathies must be carefully excluded, particularly neoplastic lesions such as lymphoma, amelanotic melanoma, and intraocular metastasis, as well as infectious etiologies including tuberculosis, syphilis, and toxoplasmosis.

In the present case, bilateral hilar lymphadenopathy together with three characteristic intraocular signs—snowball vitreous opacities, retinal periphlebitis with candle-wax drippings, and an optic disc granuloma—fulfilled the revised IWOS criteria for presumed ocular sarcoidosis (41). The pulmonary histopathologic findings of chronic granulomatous inflammation with multinucleated giant-cell reaction and no definite necrosis provided further supportive evidence for the diagnosis.

A distinctive feature of the present case is the presence of prominent vessels overlying the optic nerve head granuloma. Although previous reports have occasionally noted fine vessels overlying the lesion, dilated surface vasculature, or fluorescein leakage, most lacked combined FFA and layer-resolved OCTA evaluation, making it difficult to determine whether these vascular findings were directly associated with the granuloma, represented displaced pre-existing disc vessels, or reflected nonspecific inflammatory vascular changes. In our case, FFA demonstrated early filling of vessels overlying the lesion with marked late leakage, while OCTA showed lesion-associated abnormal flow signals that disappeared in parallel with lesion regression after treatment. However, projection artifacts and segmentation limitations cannot be fully excluded. Taken together, these findings suggest an association between the observed vascular features and inflammatory lesion activity.

Microvascular studies of pulmonary sarcoid granulomas indicate that most such lesions can be considered ‘avascular’, with blood capillaries largely confined to the outer edge of the peripheral fibrous ring (42). Whether this pattern also applies to optic nerve head granulomas, however, remains unclear in the absence of direct evidence. In the present case, we propose the hypothesis that the prominent vessels overlying the optic nerve head granuloma may have arisen from two potentially synergistic processes: (1) local release of inflammatory mediators associated with the granulomatous inflammation and (2) local ischemia and hypoxia of the disc microcirculation caused by the space-occupying effect of the lesion. Together, these factors may have promoted neovascular growth from the adjacent optic nerve head, with subsequent extension of these vessels over the surface of the granuloma. Notably, after systemic corticosteroid therapy, the lesion decreased in size in parallel with gradual disappearance of the abnormal flow signals on OCTA. We therefore speculate that the prominent vessels overlying the lesion may primarily reflect inflammation-related vascular changes.

With respect to optic disc neovascularization associated with sarcoidosis, Doxanas et al. (43) reported a patient with bilateral neovascularization of the optic nerve heads. Goldberg and Newell (4) also described a case in which new vessels at the margin of the lesion extended anteriorly beyond the edge of the optic nerve head granuloma at presentation.

Another clinically important point in this case is that, despite preserved visual acuity throughout the disease course, the optic nerve head granuloma, vitreous inflammation, and retinal vasculitis all showed clear progression over the several months during which treatment was initially declined. These observations suggest that preserved central visual acuity alone may not fully reflect ongoing ocular inflammatory activity and may support consideration of treatment even when visual acuity remains good.

## Limitations

This report has several limitations. First, as a single-case observation, the findings may not be generalizable. Second, although OCTA demonstrated lesion-associated abnormal flow signals that progressively disappeared in parallel with lesion regression, OCTA alone cannot definitively establish the histologic nature or precise origin of these signals. In particular, projection artifacts, motion artifacts, and segmentation errors cannot be fully excluded, especially in the presence of altered optic nerve head anatomy.

## Conclusion

Sarcoid optic nerve head granuloma is a rare but highly suggestive ocular manifestation that may be the presenting sign of systemic sarcoidosis and can mimic neoplastic or infectious optic disc mass lesions. The distinctive feature of the present case was the serial demonstration of lesion-associated abnormal blood flow by FFA and OCTA, with these signals progressively disappearing in parallel with lesion regression over long-term follow-up. Ultimately, the lesion regressed completely, leaving only a thin gliotic membrane, while good visual acuity was preserved. Based on our summary of previously reported cases, sarcoid optic nerve head granulomas predominantly present unilaterally and generally show varying degrees of regression following appropriate therapy. Our review of previously reported cases raises the hypothesis that visual outcomes may be more closely related to associated posterior segment inflammation and severe complications than to the granuloma itself. Multimodal imaging is helpful not only for early recognition but also for longitudinal assessment of lesion activity and treatment response. As a noninvasive modality, OCTA may be particularly useful for detecting and monitoring lesion-associated abnormal flow signals.

## Data Availability

The original contributions presented in the study are included in the article/Supplementary material, further inquiries can be directed to the corresponding author.
