# Supplementary material for: Multimodal imaging and clinical evolution of an optic nerve head granuloma with prominent overlying vessels in ocular sarcoidosis: a case report and literature review
Source: Front Neurol. 2026 Oct 15;17:1978059. doi: 10.3389/fneur.2026.1978059 (PMC13716666; doi:10.3389/fneur.2026.1978059)
Supplement: Supplementary file 1 [file Table_1.docx]

Supplementary Table 1. Previous case reports and case series of sarcoid optic nerve head granuloma.

| **Authors (Year)** | **Age / Sex / Race** | **Laterality of Granuloma** | **Prior Sarcoidosis Diagnosis** | **Initial VA** | **Concomitant Ocular Findings** | **Extraocular Clinical Features** | **Treatment** | **Length of Follow-up** | **Granuloma Outcome** | **Final VA** |
| --- | --- | --- | --- | --- | --- | --- | --- | --- | --- | --- |
| Goldberg and Newell (4)  (1944) | 23/M/Black | Unilateral | Yes | HM | Vitreous cells and pigment; retinal hemorrhages with superior temporal vein thrombosis; disc neovascularization; arteriolar attenuation; macular edema | Nontender parotid/lacrimal gland enlargement; generalized painless lymphadenopathy; bilateral hilar lymphadenopathy | None | NR | Stability | NR |
|  | 24/M/Black | Unilateral | No | 20/50 | Subhyaloid, perimacular, and peripheral hemorrhages; retinal venous irregularity and attenuation | Parotid gland enlargement; generalized lymphadenopathy | None | 5 months | Stability | 20/30 |
| Laval (5) (1952) | 48/F/White | Unilateral | No | PL | AC cells, posterior synechiae, iris pigment dispersion; peripapillary vascular engorgement, retinal hemorrhages, RNFL edema, retinal and choroidal nodules | None | None | NR | NR | PL |
| Bruntse (6) (1958) | 35/M | Bilateral | No | 6/6 (OU) | OU: pigmented KPs, iris nodules; retinal venous engorgement | Hilar lymphadenopathy | Corticotropin | 1 year | NR | 6/18 (OD); "normal" (OS) |
| Ingestad and Stigmar (7) (1971) | 26/M | Unilateral | No | 0.1 | Dense opacities on the posterior hyaloid with funnel-shaped PVD; macular edema; peritumoral retinal folds, hemorrhages, and exudates; venous congestion, peripheral vascular sheathing, candle-wax drippings | Pulmonary infiltrates, hilar lymphadenopathy; parotid gland enlargement; diabetes insipidus | Prednisolone | 20 months | Progression | NPL |
| Laties and Scheif (8) (1972) | 25/F/Black | Unilateral | No | 6/6 | Perilesional flame-shaped hemorrhages; whitish deposits on peripapillary veins | Pulmonary interstitial fibrosis, hilar lymphadenopathy | None | 19 months | Complete regression | 6/6 |
| Jampol et al. (9) (1972) | 29/F/Black | Bilateral | Yes | 20/20 (OU) | OU: peripapillary retinal folds, distended retinal veins; OS: a single retinal hemorrhage | Left pulmonary infiltrate; cervical and hilar lymphadenopathy | Prednisone | 3 months | Complete regression | NR |
| Kelley and Green (10) (1973) | 31/M/Black | Unilateral | Yes | PL | New vessels, peripapillary choroiditis; subsequent uveitis, synechiae, cataract, glaucoma | Headaches, left lateral rectus palsy, left-sided sensorineural hearing loss; parotid gland enlargement, cervical and hilar lymphadenopathy | Steroid | 16 years | NR | NPL and  enucleated |
| Turner et al. (11) (1975) | 29/F/White | Bilateral | Yes | 6/12 (OD); 6/9 (OS) | OU: vitreous flare, dilated capillaries overlying the optic disc lesion, retinal venous engorgement, candle-wax drippings; OD: cotton wool spots, hemorrhages, peripheral venous sheathing, macular edema; OS: two small discrete retinal lesions, nerve fiber bundle defects | Bilateral facial nerve palsy, peripheral neuropathy; bilateral hilar lymphadenopathy; transient papular eruption of the legs; polyarthralgia, knee effusions | Prednisone then dexamethasone | 9 months | NR | NR |
| Burns (12) (1976) | 22/F/Black | Unilateral | No | 20/200 | Localized exudative retinal detachment, subretinal hemorrhages | Amenorrhea, polydipsia, polyuria, polyphagia; skin nodules; supraclavicular and hilar lymphadenopathy | Prednisone | 4 months | NR | 20/15 |
| Gass and Olson (13) (1976) | 41/M | Bilateral | Yes | 20/25 (OD); NPL (OS) | OD: vitreous cells, candle-wax drippings, BRVO with hemorrhages, preretinal nodules; OS: history of iridocyclitis, old keratic precipitates, posterior synechiae, cataract, ciliary body detachment, granulomatous retinitis, retinal detachment | Epilepsy, ataxia, nystagmus, personality change, right-sided facial weakness, skin nodules, and an abnormal chest radiograph | Triamcinolone | 20 months | Progression (OD); NR (OS) | 20/80 (OD); NPL (OS) |
| Lustgarten et al. (14) (1983) | 32/F/Black | Unilateral | No | 20/30 | Optic disc edema, papillophlebitis; subsequent CRVO, increased granuloma size, and vitreous opacities | None | Prednisone | 13 months | NR | NPL |
| Beardsley et al. (15) (1984) | 23/M | Unilateral | No | 20/200 worsening to 20/400 | Vitreous opacities; peripapillary retinal detachment, perivenous exudates, cotton-wool spots | Bilateral hilar and peritracheal lymphadenopathy | Prednisone | 9 months | Partial regression | 20/20 |
|  | 16/M | Unilateral | No | HM | Vitreous opacities, peripapillary serous retinal detachment, perivascular exudates; subsequent anterior uveitis, secondary glaucoma | Hilar lymphadenopathy | Periocular triamcinolone and prednisone | 9 months | NR | NPL and enucleated |
|  | 24/F | Unilateral | No | 20/20 | AC cells and flare; perivascular retinal exudates with overlying vitreous debris | Malar skin eruption | Prednisone and isoniazid | 18 months | Complete regression | 20/15 |
|  | 33/M | Bilateral | No | 20/25 (OU) | None | Diffuse interstitial pulmonary markings, hilar and right paratracheal lymphadenopathy; hepatosplenomegaly | Prednisone | 4 months | NR | 20/40 (OU) |
| Karma and Mustonen (16) (1985) | 42/F | Unilateral | Yes | 1.0 | Nodular uveitis, iris granulomas; vitreous opacities; dilated leaking retinal capillaries, nodular lesions on inferotemporal vein and along scar margins of healed peripheral granulomas, macular choroidal granuloma | Purple skin plaques, joint symptoms, liver dysfunction, and hilar lymphadenopathy | Prednisone | 3 years | Partial regression | 0.1 |
|  | 35/F | Unilateral | Yes | 1.0 | Lacrimal gland enlargement, KPs, peripupillary iris atrophy; vitreous cells, snowball-like opacities; candle-wax exudates, inferoperipheral chorioretinitic lesions, serous retinal detachment | Epilepsy, lupus pernio, submandibular gland enlargement, pulmonary infiltration, and hilar lymphadenopathy | Prednisone | 2 years | Progression | CF |
| Katz et al. (17) (1991) | 56/F | Unilateral | No | 20/50 | Optic disc pseudodrusen, peripapillary retinal fullness | Middle cranial fossa gadolinium-enhancing mass on MRI | Corticosteroids | NR | NR | NR |
| Sivakumar and Chee (18) (1998) | 41/M/Indian | Unilateral | No | 6/18 | RAPD, KPs, AC cells and flare, retrolental cells, vitritis, snowballs, and focal retinal vasculitis with perivascular sheathing | Hilar lymphadenopathy | IVMP followed by prednisolone | 2 years | Complete regression | 6/6 |
| Farr et al. (19) (2000) | 31/F/Black | Unilateral | No | 20/25 worsening to 20/400 | Conjunctival nodules and hemorrhages peripheral to the granuloma | Hilar lymphadenopathy | Prednisone | 26 months | Complete regression | 20/25 |
| Frohman et al. (20) (2003) | 50/F | Unilateral | No | NPL | Periphlebitis and macular exudate | NR | NR | NR | NR | NR |
|  | 30/F | Bilateral | No | 20/20 (OU) | OU: Busacca nodules | NR | NR | NR | NR | NR |
|  | 32/F | Unilateral | No | 20/70 | Lacrimal gland enlargement, ptosis, uveitis; vitreous snowballs; optic disc shunt vessels | NR | NR | NR | NR | NR |
| Ismail et al. (21) (2005) | 40/M/Malay | Unilateral | No | 6/24 | RAPD; AC reaction; vitritis, vitreous snowballs, vitreous strands; retinal vascular tortuosity, branch retinal vein sheathing, multiple peripheral choroidal lesions | ^67^Gallium uptake in the right lacrimal gland with a “panda sign” | Prednisolone and topical dexamethasone | NR | Partial regression | 6/6 |
| Al-Jamal and Kivelä (22) (2008) | 27/M | Unilateral | No | 20/60 worsening to CF | Vitreous opacities; exudative retinal detachment, lipid exudation | Bilateral pulmonary infiltrates, mediastinal lymphadenopathy | IVMP followed by prednisolone | 1 year | Stability | 0.1 |
| Jafferji and Biswas (23) (2008) | 49/F | Unilateral | No | 6/24 | Pars plana exudates; hard exudates, optic nerve head vascular tortuosity with hemorrhages | None | IVMP followed by prednisolone and MTX | 24 months | Complete regression | 6/5 |
| Koczman et al. (24) (2008) | 24/M/Black | Unilateral | No | 20/25 | Keratoconjunctivitis sicca and panuveitis | Hilar and mediastinal lymphadenopathy; testicular involvement | IV corticosteroids then prednisone and MTX | NR | NR | 20/15 |
| Moschos and Guex-Crosier (25) (2008) | 19/M/Black | Bilateral | No | 1.0 (OD); 0.8 (OS) | OD: choroidal granuloma; OS: KPs, iris nodule; multifocal choroidal lesions | Hilar lymphadenopathy | NR | NR | NR | NR |
| Ganesh and Kaduskar (26) (2015) | 49/F | Unilateral | No | 6/24 | Optic disc hyperemia, optic disc edema, peripapillary flame-shaped hemorrhages | Hilar lymphadenopathy | IVMP followed by oral steroids and MTX | 4 years | Complete regression | 6/6 |
|  | 49/F | Unilateral | No | 6/24 | Optic disc hyperemia, optic disc edema, peripapillary retinal venous engorgement | HRCT suggestive of sarcoidosis | IVMP followed by oral steroids | 3 months | NR | 6/9 |
|  | 48/F | Unilateral | No | CF | RAPD; optic disc hyperemia, disc edema, macular star | Headache; frontoparietal subcortical vasculitis | IVMP followed by oral steroids and MTX | 5 months | NR | 6/9 |
|  | 36/M | Unilateral | No | 6/9 | RAPD; peripapillary subretinal fluid | None | Prednisolone and MTX | 7 months | Partial regression | 6/9 |
| Patterson et al. (27) (2016) | 26/M/Black | Unilateral | No | HM | RAPD; anterior segment inflammation; peripapillary exudative retinal detachment, diffuse vascular sheathing, mid-peripheral choroidal granuloma | Bilateral submandibular, supraclavicular, hilar lymphadenopathy; diffuse pulmonary nodular densities | IV corticosteroids followed by prednisone taper and adalimumab | 21 months | Partial regression | HM |
| Goldberg et al. (28) (2016) | 41/F | Unilateral | Yes | CF | Optic nerve and adjacent choroidal infiltration, peripapillary retinal thickening, ellipsoid zone attenuation | NR | NR | NR | NR | NR |
|  | 38/M | Unilateral | No | 20/50 | Vitritis; cotton wool spots, retinal edema | NR | IVMP followed by prednisone | 6 weeks | Partial regression | 20/20 |
| Hickman et al. (29) (2016) | 57/F/White | Unilateral | Yes | 6/24 | Vitreous cells; intraretinal edema, retinal inflammatory infiltrate, subretinal fluid | Vertigo and hilar lymphadenopathy | IVMP followed by prednisolone and MTX | 14 months | Complete regression | 6/9−2 |
| Padhy and Kumar (30) (2019) | 41/M | Unilateral | No | 6/24 | Vitritis; vascular sheathing, flame-shaped hemorrhages, hard exudates, posterior pole ILM folds | Bilateral hilar lymphadenopathy | Prednisolone | 6 months | Partial regression | 6/9 |
| Chung et al. (31) (2019) | 40/M | Unilateral | No | 20/200 | Vitreous hemorrhage, vitritis; subretinal fluid | Bilateral hilar lymphadenopathy | Prednisolone and IVT DEX implant | 6 months | Partial regression | 20/60 |
| Bypareddy et al. (32) (2019) | 17/M | Unilateral | No | 6/60 | Superior quadrant venous engorgement, flame-shaped hemorrhages, macula-involving peripapillary serous retinal detachment | None | Prednisolone | 2 years | Complete regression | 6/6 |
|  | 18/M | Unilateral | No | 6/60 | Vitritis; optic disc edema, hyperemia, hemorrhages; peripapillary venous engorgement, retinal phlebitis; macular edema, serous macular detachment | None | Prednisolone | Over a year | Complete regression | 6/9 |
| Krishna et al. (33) (2020) | 61/M | Unilateral | Yes | NPL | Neovascular glaucoma, rubeosis iridis, anterior synechiae; attenuated retinal vessels | History of lymphoma; generalized lymphadenopathy (hilar, mediastinal, axillary, iliac, inguinal); bilateral sub-central pulmonary nodules | Prednisolone and infliximab | NR | NR | NPL and enucleated |
| Kumar et al. (34) (2021) | 45/F/Indian | Unilateral | No | CF | Vitritis; epilesional dilated capillaries, microaneurysms; superficial hemorrhages, superotemporal BRVO; macular ischemia | Bilateral hilar lymphadenopathy | Prednisolone and azathioprine | 3 months | Partial regression | CF |
| Mahmoudzadeh et al. (35) (2022) | 76/F/White | Unilateral | No | 20/400 | Peripheral retinal hypopigmented lesions, peripheral arteriolar attenuation; posterior macroaneurysms, flame-shaped hemorrhages; peripapillary outer retinal atrophy, shallow pigment epithelial detachments | Fatigue, weight loss, dry cough, arthralgia; complete AV block; bilateral hilar lymphadenopathy; hypermetabolic mediastinal, hepatic, splenic, skeletal nodules | IVMP followed by prednisone → MTX → infliximab | NR | NR | 20/100 |
| Oyeniran et al. (36) (2024) | 59/F/White | Unilateral | No | 20/20 | Optic disc elevation, epipapillary vascular tortuosity, intraretinal hemorrhages | PET-avid mediastinal and bilateral hilar lesions | Prednisone + MTX → adalimumab → oral DEX → rituximab | NR | Complete regression | NR |
| Babu and Mooss (37) (2024) | 55/M | Unilateral | No | 6/9 | None | Pretracheal, precarinal lymphadenopathy; bilateral perilymphatic pulmonary nodules | IVMP → oral steroids + MTX → oral steroids + MMF + IVT DEX implant | 22 months | Complete regression | 6/9 |
| Saidane et al. (38) (2024) | 62/F | Unilateral | No | 6/10 | Granulomatous KPs, AC cells; vitreous haze, inferior snowbanking; peripapillary and macular SRF, inferotemporal perivascular inflammatory lesions | 3-mm intraorbital nodule at left optic nerve origin; bilateral hilar lymphadenopathy | Oral steroids and MTX | 1 month | Partial regression | NR |
| Soifer et al. (39) (2025) | 36/M/White | Bilateral | No | 20/16 (OD); 20/20 (OS) | OU: peripapillary and posterior birdshot-like choroidal lesions | Hypermetabolic mediastinal lymphadenopathy | Prednisone and MMF | 3 months | Partial regression | NR |
| Present case | 31/F/Chinese | Unilateral | No | 20/20 | Vitreous snowballs; retinal vasculitis, candle-wax exudates | Bilateral pulmonary nodules; mediastinal and bilateral hilar lymphadenopathy | IVMP followed by prednisone | 20 months | Complete regression | 20/20 |

**Note.** Visual acuity and treatment are reported as described in the original publications.

**Abbreviations:** AC = Anterior Chamber; AV = Atrioventricular; BRVO = Branch Retinal Vein Occlusion; CF = counting fingers; CRVO = central retinal vein occlusion; DEX = Dexamethasone; HM = perception of hand movements; HRCT = High-Resolution Computed Tomography; ILM = Internal Limiting Membrane; IV = intravenous; IVMP = Intravenous methylprednisolone; IVT = Intravitreal; KPs = Keratic Precipitates; MMF = Mycophenolate Mofetil; MRI = magnetic resonance imaging; MTX = Methotrexate; NPL = no perception of light; NR = Not Reported; OD = right eye; OS = left eye; OU = both eyes; PET = Positron Emission Tomography; PL = perception of light; PVD = Posterior Vitreous Detachment; RAPD = relative afferent pupillary defect; RNFL = Retinal Nerve Fiber Layer; SRF = Subretinal Fluid; VA = Visual Acuity.
